# A Comparison of Treatment Options for Type 1 and Type 2 Caesarean Scar Pregnancy: A Retrospective Case Series Study

**DOI:** 10.3389/fmed.2021.671035

**Published:** 2021-06-15

**Authors:** Fanghua Shen, Hongdao Lv, Liming Wang, Ruiheng Zhao, Mancy Tong, Arier Chi-Lun Lee, Fang Guo, Qi Chen

**Affiliations:** ^1^Department of Obstetrics and Gynaecology, Suzhou Ninth People's Hospital, Suzhou, China; ^2^Department of Obstetrics, Gynaecology and Reproductive Sciences, Yale School of Medicine, New Haven, CT, United States; ^3^Section of Epidemiology and Biostatistics, School of Population Health, The University of Auckland, Auckland, New Zealand; ^4^The Hospital of Obstetrics and Gynaecology, Fudan University, Shanghai, China; ^5^Department of Obstetrics and Gynaecology, The University of Auckland, Auckland, New Zealand

**Keywords:** caesarean scar pregnancy, surgical treatment, medical treatment, subtype of CSP, a success rate

## Abstract

**Background:** There is currently no agreement on the optimal management of caesarean scar pregnancy. Caesarean scar pregnancy is currently categorised into two subtypes according to the site of implantation. This may consequently result in the difference in treatment options. However, the comparison of the success rate of each treatment option according to the subtypes has not been fully investigated.

**Methods:** 71 patients who were treated by uterine curettage (D and C), or uterine artery embolization with curettage (UAE) or hysteroscopy in conjunction with laparoscopy between January 2016 and March 2020 were included. Data on maternal age, gestational sac age, the sac diameter, the interval between two pregnancies, the number of previous caesarean sections, amount of bleeding and β-hCG levels were collected and analysed dependent on the subtypes.

**Results:** There was no difference in the clinical parameters of the cases who received different options of treatment, as well as no difference in the clinical parameters between type 1 and type 2 caesarean scar pregnancy. The primary success rate for type 1 caesarean scar pregnancy by D and C, or UAE, or hysteroscopy in conjunction with laparoscopy was 95, or 100 or 100%, respectively. The primary success rate for type 2 caesarean scar pregnancy by D and C, or UAE, or hysteroscopy in conjunction with laparoscopy was 27, or 67, or 95% respectively.

**Conclusion:** Our data demonstrates that hysteroscopy in conjunction with laparoscopy for type 2 caesarean scar pregnancy was the most successful compared to other options, but for type 1 caesarean scar pregnancy, D and C could be the cost-effective option.

## Highlights

1. Whether the subtypes of caesarean scar pregnancy differ to the treatment options has not been well-studied.
2. For type 1 caesarean scar pregnancy, there was no difference in the treatment options.
3. For type 2 caesarean scar pregnancy, hysteroscopy in conjunction with laparoscopy was the most successful.

## Introduction

Caesarean scar pregnancy is a relatively new type and rare form of ectopic pregnancy at the site of the scar from a previous caesarean section, resulting in implantation of a blastocyst within myometrial scar tissue in the anterior lower uterine segment (1). The estimated incidence of caesarean scar pregnancy is regionally dependent. A recent study reported that the estimated incidence of caesarean scar pregnancy was 1.5 per 10,000 pregnancies in the United Kingdom (2). While a systemic review study reported that the estimated incidence of caesarean scar pregnancy was 1 per 1,800 to 2,600 pregnancies globally, which represents 6% of all ectopic pregnancies in women with prior caesarean delivery (3).

Given the risk of life-threatening complications, management of caesarean scar pregnancy is another challenge for gynaecologists. Early treatment has benefits to prevent maternal complications, as caesarean scar pregnancy is a precursor of abnormally adherent placentae in the second and third trimesters of pregnancy (4). Medical treatment including systemic or local methotrexate (MTX) with or without uterine curettage (D and C), and surgical treatment including uterine artery embolization (UAE) or hysteroscopy in conjunction with or without laparoscopy or other different combinations are commonly used in clinical practices worldwide. However, to date there is no agreement on the most optimal management of caesarean scar pregnancy, due to the limited number of clinical studies (including clinical trials) with a large sample size (5, 6). The effectiveness of medical vs. surgical treatments on caesarean scar pregnancy is controversial (2, 7).

Caesarean scar pregnancy is now categorised into two different subtypes according to the site of implantation. Type 1 caesarean scar pregnancy is when it develops in the myometrium and grows toward the cervico-isthmic space or uterine cavity, while type 2 caesarean scar pregnancy is when there is progression towards the bladder and abdominal cavity (6, 8, 9). The two subtypes of caesarean scar pregnancy could manifest into different complications. For example, uterine rupture occurs more often in type 2 (8, 9). Although currently expectant, medical and surgical treatment is recommended by RCOG/AEPU Green-top Guideline for selective abortion (10), there is currently no general agreement on the optimal management of type 1 and type 2 caesarean scar pregnancy (11). Studies about the comparison of different treatment options considering the subtype of caesarean scar pregnancy have not been well-investigated.

In this retrospective study, we aimed to compare the primary treatment options of caesarean scar pregnancy on its success rate, that is currently performed in our hospital, taking the subtypes into account. In addition, we also investigated whether there is an association between optimal treatment options and clinical parameters including serum levels of β-hCG, bleeding during treatment and gestational sac age or size.

## Methods

This retrospective study was approved by the Ethics Committee of Suzhou Ninth People's Hospital, Jiangsu Province of China (ref: 2019-008).

### Study Population

During January 2016 to March 2020, a total number of 71 patients who were diagnosed with caesarean scar pregnancy in our hospital were included in this study. Of these cases, 27 patients were diagnosed with type 1 caesarean scar pregnancy, and 44 patients were diagnosed with type 2 caesarean scar pregnancy. Overall, 30 patients primarily received treatment with systemic or local MTX with or without uterine curettage, 17 patients primarily received the treatment of uterine artery embolization with uterine curettage, and 24 patients primarily received the treatment of hysteroscopy in conjunction with laparoscopy. The decision for treatment methods was dependent on the experiences of an individual gynaecologist.

Data on maternal age, parity, gravida, gestational sac age at diagnosis, the size of sac, the interval between this pregnancy and last caesarean section, the number of previous caesarean section, the amount of bleeding during the treatment and the serum levels of β-hCG at diagnosis were collected from the hospital electronic database and analysed.

The diagnosis of caesarean scar pregnancy was based on findings from the transvaginal ultrasound image including the presence of a gestational sac in the area of the scar, in addition to a history of prior caesarean section and positive pregnancy test. Subtypes of caesarean scar pregnancy were based on the site of sac implantation described by previous studies (8, 9). The success rate was calculated by the primary treatment, without additional treatment. The criteria of success mainly include (1) the decline of serum levels of β-hCG, and (2) the absence of the gestational sac by ultrasound after treatment. For patients who failed the primary option, UAE or hysteroscopy in conjunction with laparoscopy was performed, and for those rare cases of failure in the option of hysteroscopy in conjunction with laparoscopy, abdominal surgery was performed to remove the gestational sac.

### Power of Sample Size

The sample size calculation was based on the estimated incidence of caesarean scar pregnancy [6% of all ectopic pregnancies in women with prior caesarean section worldwide (3, 12)]. At least 62 cases were needed for a statistical power of 90% for each group to detect a significant difference between two groups at a level of 0.05. In this study 71 cases were included.

### Statistical Analysis

Data were presented as median and range or percentage, as appropriate. The statistical differences in maternal age, gestational sac age or size at diagnosis, the interval between this pregnancy and last caesarean section, serum levels of β-hCG, and amount of bleeding were assessed by the Mann-Whitney *U*-test or ANOVA test, when it appropriated using the Prism software package. Multiple logistic regression was used to analyses the probability of treatment success depending on the method of treatment, a subtype of cesarean scar pregnancy and maternal age using SAS for Windows 9.4 (SAS Institute Inc., Cary, NC). Analyses were two-tailed with a significance level of 0.05.

## Results

Overall, the median age of patients was 31 (range 19–42) years and median gestational sac age was 50 (range 34–90) days. The median interval between this pregnancy and the last caesarean section was 5 (0.8–19) years. A total of 27 (38%) patients were diagnosed with type 1 and 44 (62%) patients were diagnosed with type 2 caesarean scar pregnancy. The overall primary success rate was 80% (57 of 71 cases). There was no difference in gestational sac age at diagnosis, size of sac, and the interval between current pregnancy and last caesarean section between cases with primary success and those who did not (Table 1). The median maternal age in cases with primary success was significantly younger (Table 1, *p* = 0.036). The median amount of bleeding during treatment was significantly higher in unsuccessful cases (Table 1, *p* < 0.001).

**Table 1:** Comparison in clinical characteristics between successful and unsuccessful treatment.

	**Successful (*n* = 57)**	**Unsuccessful (*n* = 14)**	***P*-value**
Maternal age (median/range)	30 (19–42)	33 (25–39)	***P*** **=** **0.036**
Gestational sac age (days, median/range)	49 (34–90)	54 (41–85)	*P* = 0.165
Sac diameter (mm, median/range)	22 (7–64)	32 (11–63)	*P* = 0.075
Interval pregnancies (years, median/range)	5 (0.8–19)	6 (2–12)	*P* = 0.295
β-hCG (IU/L, median/range)	36,780 (142–146,982)	57,850 (2,098–177,245)	*P* = 0.355
Bleeding (ml, median/range)	30 (10–1,000)	550 (20–1,400)	***P*** **< 0.001**

*The bold values in indicate the statistical difference*.

We next analysed the clinical characteristics between type 1 and type 2 caesarean scar pregnancy. There was no difference in the maternal age, gestational sac age at diagnosis, size of sac, the interval between this pregnancy and last caesarean section, and amount of bleeding during treatment between type 1 and type 2 caesarean scar pregnancy (Table 2). But serum levels of β-hCG at diagnosis in type 1 cases was significantly higher than that in type 2 caesarean scar pregnancy (Table 2, *p* = 0.014). We then compared the clinical parameters of cases with primary success between type 1 and type 2 caesarean scar pregnancy. Again, there was no difference in the maternal age, gestational sac age at diagnosis, size of sac, the interval between this pregnancy and last caesarean section, and amount of bleeding during treatment between the two groups (Table 3). However, serum levels of β-hCG at diagnosis in type 1 caesarean scar pregnancy with primary success was significantly higher than type 2 caesarean scar pregnancy with success (Table 3, *p* = 0.006).

**Table 2:** Clinical characteristics according to the subtypes of caesarean scar pregnancy.

	**Type 1 (*n* = 27)**	**Type 2 (*n* = 44)**	***P*-value**
Maternal age (median/range)	30 (19–42)	31 (20–40)	*P* = 0.588
Gestational sac age (days, median/range)	46 (34–72)	52 (39–90)	*P* = 0.229
Sac diameter (mm, median/range)	21 (16–51)	28 (7–64)	*P* = 0.477
Interval pregnancies (years, median/range)	5 (1–16)	5 (0.8–19)	*P* = 0.997
β-hCG (IU/L, median/range)	52,360 (558–147,124)	30,692 (142–177,245)	***P*** = **0.014**
Bleeding (ml, median/range)	30 (20–500)	100 (10–1,400)	*P* = 0.045

*The bold values in indicate the statistical difference*.

**Table 3:** Comparison in clinical characteristics of successful cases according to subtypes of caesarean scar pregnancy.

	**Type 1 (*n* = 26)**	**Type 2 (*n* = 31)**	***P*-value**
Maternal age (median/range)	30 (19–42)	31 (20–40)	*P* = 0.722
Gestational sac age (days, median/range)	46 (34–72)	50 (39–90)	*P* = 0.461
Sac diameter (mm, median/range)	21 (16–51)	25 (7–64)	*P* = 0.991
Interval pregnancies (years, median/range)	5 (1–16)	5 (0.8–19)	*P* = 0.832
β-hCG (IU/L, median/range)	50,292 (558–147,124)	27,196 (142–126,656)	***P*** = **0.006**
Bleeding (ml, median/range)	30 (20–200)	30 (10–100)	*P* = 0.445

*The bold values in indicate the statistical difference*.

The overall primary success rate by uterine curettage treatment, or uterine artery embolization with curettage, or hysteroscopy in conjunction with laparoscopy was 70, or 76, or 96% respectively. There was no difference in the maternal age, gestational sac age at diagnosis, size of sac, numbers of previous caesarean section, and the interval between this pregnancy and last caesarean section among the three different treatment options (Table 4). We next analysed the primary success rate by different options of treatment, taking into account subtypes of caesarean scar pregnancy (Table 5). In type 1 caesarean scar pregnancy, the success rate by uterine curettage treatment, or uterine artery embolization with curettage, or hysteroscopy in conjunction with laparoscopy was 95, or 100, or 100%, respectively. In type 2 caesarean scar pregnancy, the success rate by uterine curettage treatment, or uterine artery embolization with curettage, or hysteroscopy in conjunction with laparoscopy was 27, or 67 or 95%, respectively. There was a significantly higher success rate in type 1 caesarean scar pregnancy that was treated by uterine curettage, compared to type 2 caesarean scar pregnancy (Table 5, *p* = 0.0002). In type 2 caesarean scar pregnancy, compared to the option of uterine curettage, hysteroscopy in conjunction with laparoscopy showed a significantly higher success rate (95 vs. 27%, *p* = 0.0001, Table 5).

**Table 4:** The differences in clinical characteristics between patients who received different treatment.

	**D and C group (*n* = 30)**	**UAE group (*n* = 17)**	**Hysteroscopy plus laparoscopy (*n* = 24)**	***P*-value (ANOVA)**
Maternal age (years, median/range)	30.5 (19–42)	31 (26–39)	31 (20–40)	*P* = 0.971
Gestational sac age (days, median/range)	48 (40–90)	53 (40–64)	50 (34–90)	*P* = 0.892
Sac diameter (mm, median/range)	22 (7–63)	28 (11–64)	26 (7–60)	*P* = 0.817
Interval pregnancies (years, median/range)	5 (1–16)	5 (2–12)	5.4 (0.8–19)	*P* = 0.957
β-hCG (IU/L, median/range)	46,024 (313–137,878)	44,630 (2,098–177,245)	32,891 (142–147,124)	*P* = 0.281
Bleeding (ml, median/range)	50 (20–1,400)	20 (10–1,200)	100 (10–1,000)	***P*** **=** **0.012**
Number of prior to caesarean section				Fisher's exact
1 (number, %)	20 (67%)	12 (70%)	10 (41%)	*P* = 0.097
2 (number, %)	10 (33%)	5 (30%)	14 (59%)	

*D and C, uterine curettage; UAE, uterine artery embolization*.

*The bold values in indicate the statistical difference*.

**Table 5:** The success rate by different treatments according to the subtypes of caesarean scar pregnancy.

	**Type 1 (*n* = 27)**	**Type 2 (*n* = 44)**	***P*-value (fisher's exact**
D and C group (%)	95% (18 out of 19 cases)	27% (3 out of 11 cases)	*P* = 0.0002
UAE group (%)	100% (5 out of 5 cases)	67% (8 out of 12 cases)	*P* = 0.2605
Hysteroscopy plus laparoscopy (%)	100% (3 out of 3 case)	95% (20 out of 21 cases)	*P* > 0.999

*D and C, uterine curettage; UAE, uterine artery embolization*.

Multiple logistic regression analysis showed that there was a statistically significant association between the success rate and the treatment options (*p* = 0.003), or the subtypes of caesarean scar pregnancy (*p* = 0.0016). However, there was some evidence of the association between success rates and sac size (*p* = 0.063). In our current study, we did not see any severe complications after treatments.

None of the patients had hysterectomies during treatment in this study. There were two patients who had a blood loss of over 1,000 ml in each treatment group. There were two patients who needed blood transfusion in the D and C or UAE group, or three patients needed blood transfusion in the hysteroscopy plus laparoscopy group. Due to the small sample size, we were not able to perform a statistical analysis in the difference of intra-treatment complications. The median time for the menstrual cycle to re-establish was 35 days (ranged from 28 to 75 days), which is normal and was not different between the three treatment groups. The levels of β-hCG quickly declined to normal levels ranging from 17 to 70 days and were not different between the three treatment groups.

## Discussion

In this retrospective and observational study, we found that the incidence of type 1 caesarean scar pregnancy was 38%, while the incidence of type 2 caesarean scar pregnancy was 62% in our study cohort. Serum levels of β-hCG at diagnosis was higher in type 1 caesarean scar pregnancy. Treatment with uterine curettage had 95% success in type 1 caesarean scar pregnancy, while this option of treatment only showed 27% success in type 2 caesarean scar pregnancy. Treatment with hysteroscopy in conjunction with laparoscopy showed a significantly higher success rate in type 2 caesarean scar pregnancy (95%), compared to uterine curettage (27%). Our findings suggest that for type 1 caesarean scar pregnancy, there was no difference in the treatment options and for type 2 caesarean scar pregnancy, hysteroscopy in conjunction with laparoscopy was the most successful.

The development of ultrasound technology has resulted in being able to distinguish between the subtypes of caesarean scar pregnancy better. Whether clinical parameters at the time of diagnosis are associated with the optimal treatment option, or whether there was a difference in clinical parameters between type 1 and type 2 caesarean scar pregnancy has not been fully investigated. A recent study reported that large gestational sac (over 10 mm) at presentation should be referred for surgical treatment (13). However, our current data only showed some evidence of the association between success rate and sac size. A recent study also suggested that treatment options should be individualised, as there is currently no evidence-based recommendations for the management of caesarean scar pregnancy due to the lack of randomised clinical trials (5). This meant that most of the studies of caesarean scar pregnancy reported in the literature were case series. In our current study, we found no difference in clinical parameters in cases who received different options of treatment, as well as no difference in clinical parameters between type 1 and type 2 caesarean scar pregnancy, although the higher levels of β-hCG were seen in type 1 caesarean scar pregnancy.

To date, studies comparing the options of primary treatment in caesarean scar pregnancy according to the subtypes are very limited. Although one study reported that treatment with hysteroscopy in conjunction with laparoscopy is more suitable for type 2 caesarean scar pregnancy, that study did not compare hysteroscopy in conjunction with laparoscopy with other options of treatment (11). Our multiple logistic regression analysis showed an association between the success rate and the primary treatment options. Treatment by uterine curettage showed a success rate of 48% [reviewed in (14)] and it should be performed when the serum β-hCG level is <50 IU/L without blood flow velocity (15) or in type 1 caesarean scar pregnancy (16). In our current study, we found that the overall success rate by uterine curettage was 70% and there was no difference in the levels of β-hCG in cases who received three different options of treatment. When we took into account the subtypes of caesarean scar pregnancy, we found an 95% success rate of uterine curettage in type 1 caesarean scar pregnancy. In contrast, only 27% success rate of uterine curettage in type 2 caesarean scar pregnancy. Taken together, our data suggest that treatment with uterine curettage may have a higher success rate in type 1 than type 2 caesarean scar pregnancy.

Uterine artery embolization is an adjuvant treatment option that can reduce bleeding especially in type 2 caesarean scar pregnancy (3). A systemic review reported that the success rate by uterine artery embolization with curettage ranged from 93 to 100% (17) [reviewed in (14)]. In our current study, we found that the overall success rate by uterine artery embolization with curettage was 76%. However, when we took into account the subtypes of caesarean scar pregnancy, we found that the success rate in type 1 caesarean scar pregnancy that was treated with uterine artery embolization with curettage was 100%. In contrast, the success rate in type 2 caesarean scar pregnancy was 67%.

Treatment with laparoscopy has shown a 97% success rate without severe complications [reviewed in (14)]. A recent study reported that treatment with hysteroscopy in conjunction with laparoscopy is more suitable for type 2 caesarean scar pregnancy (11). In our current study, we found that 48% (20 out of 44 cases) of type 2 caesarean scar pregnancy were treated with hysteroscopy in conjunction with laparoscopy, suggesting that gynaecologists in our hospital are likely to treat type 2 caesarean scar pregnancy with this option (11). The success rate of hysteroscopy in conjunction with laparoscopy reached to 95% in type 2 caesarean scar pregnancy, which was significantly higher than treatment with uterine curettage, and higher than treatment with uterine artery embolization.

In our current study, we did not see any severe complications after treatments. None of the patients had hysterectomies, only six patients (8%) had bleeding over 1,000 ml and only seven patients (9.8%) needed a blood transfusion. The median time for the menstrual cycle to re-establish was 35 days (ranged from 28 to 75 days), which is normal. The levels of β-hCG quickly declined to normal levels ranging from 17 to 70 days.

Currently, expectant, medical and surgical treatment is recommended by RCOG/AEPU Green-top Guideline for selective abortion (10). Non-surgical management is a more common option in western countries such as the United States of America (7, 14). In addition, a recent study reported that the necessity of UAE is low (18), suggesting that the UAE may be overestimated in clinical practice. Therefore, in our hospital, the D and C is the first option of treatment for caesarean scar pregnancy.

There are some limitations in this study. Firstly, the data were collected from a local hospital which may not be representative of the whole to the Chinese population. Furthermore, there is a relatively small sample size in this study, in particular in each treatment group. This limitation also does not allow us to estimate the incidence of caesarean scar pregnancy in order to compare the incidence of caesarean scar pregnancy between countries. Secondly, only two surgical treatment methods are performed in our clinical practice, so we are not in the position to compare other surgical treatment methods. Lastly, as there is currently no standard protocol for the management of caesarean scar pregnancy, this means the decision for which treatment method is chosen is dependent on an individual gynaecologist's experiences in our hospital. This could induce a bias in the success rate reported in this study. However, we are currently performing a randomised clinical trial to investigate whether the treatment option(s) is associated with complications during and post-treatment (19).

## Conclusion

To our knowledge, this is the first study to compare the primary success rates of different treatment options for caesarean scar pregnancy, considering subtype of the disease. Our study demonstrates that clinical parameters are not associated with the success by treatment options between type 1 and type 2 caesarean scar pregnancy. In addition, our data found that there was no difference in the primary success rate between medical and surgical treatments in type 1 caesarean scar pregnancy. However, for type 2 caesarean scar pregnancy, treatment with hysteroscopy in conjunction with laparoscopy seems more likely to be successful. However, individualised care and management are paramount depending on patient profile, hospital protocol and individual gynaecologists' experiences.

## Data Availability Statement

The raw data supporting the conclusions of this article will be made available by the authors, without undue reservation.

## Ethics Statement

This retrospective study was approved by the Ethics Committee of Suzhou Ninth People's Hospital, Jiangsu Province of China (ref: 2019-008). The patients/participants provided their written informed consent to participate in this study.

## Author Contributions

FS and RZ: collected the data reported in this work. HL, LW, FG, and QC: contributed to conception and design of this study. AL: contributed to data analysis. MT and QC: wrote the manuscript draft. All authors were involved in the drafting, editing, and approval of the manuscript for publication.

## Conflict of Interest

The authors declare that the research was conducted in the absence of any commercial or financial relationships that could be construed as a potential conflict of interest.
